# Hybrid Method Based on Information Gain and Support Vector Machine for Gene Selection in Cancer Classification

**DOI:** 10.1016/j.gpb.2017.08.002

**Published:** 2017-12-12

**Authors:** Lingyun Gao, Mingquan Ye, Xiaojie Lu, Daobin Huang

**Affiliations:** School of Medical Information, Wannan Medical College, Wuhu 241002, China

**Keywords:** Gene selection, Cancer classification, Information gain, Support vector machine, Small sample size with high dimension

## Abstract

It remains a great challenge to achieve sufficient **cancer classification** accuracy with the entire set of genes, due to the high dimensions, small sample size, and big noise of gene expression data. We thus proposed a hybrid **gene selection** method, **Information Gain-Support Vector Machine** (IG-SVM) in this study. IG was initially employed to filter irrelevant and redundant genes. Then, further removal of redundant genes was performed using SVM to eliminate the noise in the datasets more effectively. Finally, the informative genes selected by IG-SVM served as the input for the LIBSVM classifier. Compared to other related algorithms, IG-SVM showed the highest classification accuracy and superior performance as evaluated using five cancer gene expression datasets based on a few selected genes. As an example, IG-SVM achieved a classification accuracy of 90.32% for colon cancer, which is difficult to be accurately classified, only based on three genes including *CSRP1*, *MYL9*, and *GUCA2B*.

## Introduction

The incidence and mortality of cancer have been increasing in recent years, posing a serious threat to human health. Uncontrolled proliferation and metastasis of cancer cells pose challenges in identification of cancer types. Moreover, most patients are diagnosed with cancer only when it is at an advanced stage [1], further increasing the difficulty in cancer treatment. DNA microarray technology is able to simultaneously evaluate the expression levels of numerous genes [2], enabling the identification of cancer types at the molecular level. However, the massive amount of data generated and unavoidable errors occurring during experimental processes pose great challenges to the analysis of gene expression data.

Gene expression data are featured with high dimensions, small sample size, and big noise, whereas only a few genes among the genes examined could play an important role in cancer prediction [3]. Therefore, various methods had been developed to select as few informative genes as possible, while maintaining high classification accuracy. Generally, gene selection approaches are divided into two categories: filter and wrapper [4]. Filter methods use feature-ranking techniques as the basis for gene selection. The top-ranked genes are chosen as informative genes. Common ranking methods include information gain (IG) [5], *t*-statistic [5], reliefF [6], and signal-to-noise ratio [7]. For instance, IG, also known as the Kullback–Leibler divergence, can analyze the correlation between attributes and classes. The wrapper methods identify a powerful gene subset according to the evaluation of a classifier, such as genetic algorithm [8], decision tree [9], and support vector machine (SVM) [10]. Filter methods generally run faster; however, they are unable to analyze the relationships among genes. Conversely, wrapper methods have better performance; however, they require great computational expenses [11]. Therefore, numerous hybrid methods have been proposed to achieve optimal performance [12], [13], [14], [15], [16].

SVM is a machine-learning algorithm based on the principle of structural risk minimization [17]. It shows superior classification performance with better global minimization and generalization ability than traditional classifiers [18]. Therefore, SVM-based methods have been commonly developed for the selection and classification of genes. For instance, Li et al. [19] used a weighted doubly regularized SVM to adaptively identify informative genes. Chan et al. [20] developed a firefly-optimized penalized SVM with SCADL2 penalty function, SVM-SCADL2-FFA, to optimize tuning parameters for the efficient identification of informative genes and pathways. Apart from the extended versions, there are also many integrated methods involving the traditional implementation of SVM [21], [22]. Briefly, SVMs can be used to solve various problems with outstanding performance in the real world.

In this study, considering the huge computational cost of SVM to handle numerous genes, we employed a hybrid method combining IG with SVM for selecting informative genes. IG was initially used to select genes in order to reduce the original data dimension, and further filtration of redundant genes was performed next using SVM. The obtained informative genes were finally evaluated by the LIBSVM classifier.

## Method

### IG

The importance of genes in a specific category can be evaluated using differences between entropy and conditional entropy, *i.e.*, IG [5], [23]. IG *g*(*Y*, *X*) indicates the reduction of uncertainty [24] as define below

(1) g(Y,X)=H(Y)-H(Y|X)

where *H*(*Y*) denotes the entropy of dataset *Y*, which quantifies the uncertainty involved in predicting the value of a random variable, whereas *H*(*Y|X*) denotes the conditional entropy, which represents the uncertainty based on the known variable *X*. *p* denotes probability distribution. *H*(*Y*) and *H*(*Y|X*) are defined as follows:

(2) H(Y)=-∑p(y)logp(y)

(3) $$H(Y|X)=\underset{x\in X}{\sum }p(x)H(Y|X=x)$$

The order of every single gene is arranged in line with the IG value, and high-ranked genes are selected as input features.

### SVM

The characteristics of gene expression data, such as small sample size and high dimensions, are well-suited for SVM. Particularly, only a few support vectors in the training set are applied for constructing the decision function that leaves the largest separate margin. By doing this, SVM obtains the optimal hyperplane, which would result in the maximal generalization ability [25].

Given training set

(4) $$T=\{(x_{i}\text{,}y_{i})|i=1\text{,}2\text{,}\cdots \text{,}m\}\text{,}\;x_{i}\in R^{n}\text{,}\;y_{i}\in \{-1\text{,}1\}$$

where *y_i_* is the label class, m is the number of examples. The main purpose of SVM is to establish the optimal hyperplane:

(5) D(x)=ω·x+b

where *ω* denotes the weight vector, and *b* denotes the bias value. When addressing nonlinear problems, SVM adopts the kernel function to map data into high-dimensional space. *C* denotes the penalty factor, and *ξ* denotes the relaxation factor. To maximize the separating margin, and minimize the training error, the objective function can be expressed as:

(6) $$\begin{array}{cc} & \min\;J=\frac{1}{2}\|\omega \;{\|}^{2}+C\overset{m}{\underset{i=1}{\sum }}\xi_{i}\\ & s.t.\;y_{i}(\omega \cdot x+b)⩾1-\xi_{i}\\ & \;\;\xi_{i}⩾0\text{,}\;i=1\text{,}2\text{,}\ldots \text{,}m \end{array}$$

The optimal solution (*ω^∗^*, *b^∗^*) about (*ω*, *b*) can be achieved using the Lagrange duality theorem and quadratic programming, thereby decision function can be calculated. *α_i_* is the Lagrange multiplier:

(7) $$\begin{array}{cc} & f(x)\;=\text{sgn}\left(\overset{m}{\underset{i=1}{\sum }}a_{i}y_{i}K(x_{i}\cdot x)+b^{*}\right)\\ & s.t.\;0⩽a_{i}⩽C \end{array}$$

Particularly, the kernel function *K*(*x_i_*, *x*) plays an important role in addressing nonlinear problems. The commonlyused functions include the linear kernel function, polynomial function, radial basis function (RBF) [26], and sigmoid kernel function. Of them, the linear kernel function is a special case of RBF. Compared with the polynomial kernel function, RBF has fewer parameters to be determined and was adopted in the current study:

(8) $$K(x_{i}\text{,}x)=\exp(-\gamma \|x_{i}-x{\|}^{2})$$

### Proposed approach

In this study, IG was applied to make a preliminary gene selection. We used the InfoGainAttributeEval and Ranker evaluation tools of WEKA to complete this process. InfoGainAttributeEval evaluates genes relevant to clinical outcomes according to IG, and Ranker ranks individual genes on the basis of evaluation outcomes.

Considering the efficiency of filters, this study also used three other filter methods including gain ratio, reliefF, and correlation. Based on IG, the gain ratio algorithm is, frequently used in decision tree C4.5. ReliefF is a feature-weighting algorithm, which assigns different weights to features based on correlation. The correlation between every single gene and the class is usually measured using the Pearson correlation coefficient, with a higher value indicating a more important gene.

**Figure 1:**
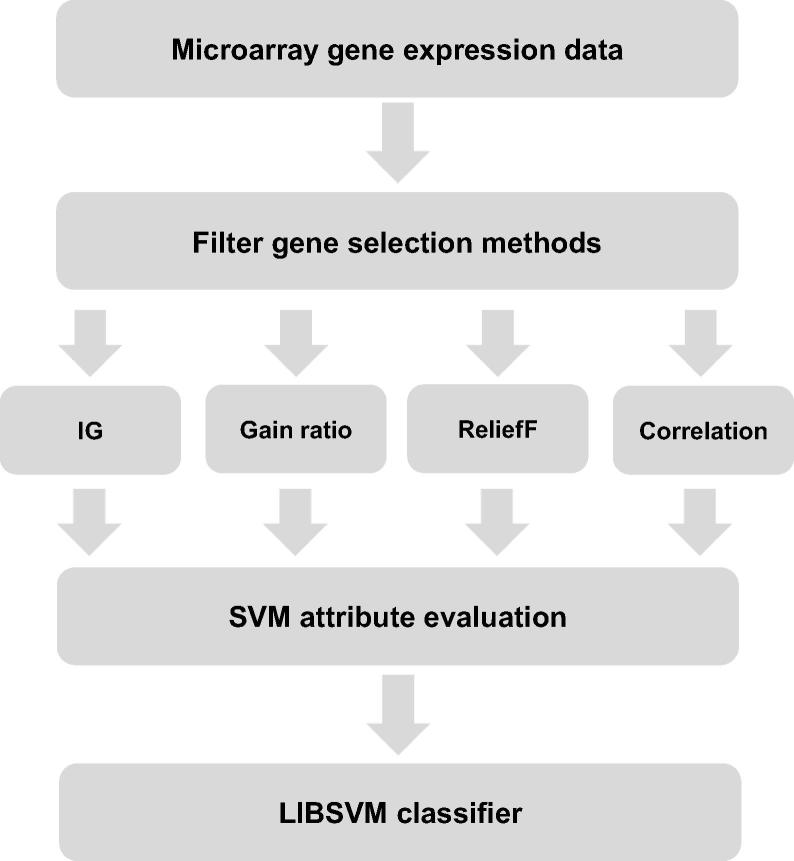
**Workflow of proposed approach**

In order to choose genes with high classification value, we combined the high efficiency of filters and the excellent performance of wrappers. In addition to the application of IG, a further feature selection algorithm, SVM, was also employed. Meanwhile, gain ratio, reliefF, and correlation attribute evaluation combined with SVM were implemented, respectively, to select genes as well. The ultimately obtained informative genes served as the input data for the LIBSVM classifier to assess classification accuracy. Because of the small sample size of gene expression data, 10-fold cross-validation was utilized for the evaluation of the selected genes. Figure 1 illustrates the schematic diagram of the proposed method.

With two stages included in the process of selecting optimal gene, the hybrid method fully utilizes different algorithms to obtain fewer informative genes and achieve better classification accuracy.

## Results and discussion

### Experimental datasets

Five benchmark microarray datasets of two classes were evaluated in this study. Among the five datasets, three were obtained from normal or cancerous tissues, which include lung cancer, colon cancer, and prostate cancer. The dataset for the diffuse large B-cell lymphoma (DLBCL) was obtained from two different DLBCL subgroups, *i.e.*, germinal center B-cell like subgroup and activated B-cell like subgroup. Similarly, the dataset for leukemia was obtained from acute myeloid leukemia (AML) and acute lymphoblastic leukemia (ALL) cases. All these datasets were downloaded from http://datam.i2r.a-star.edu.sg/datasets/krbd/. The detailed description is provided in Table 1.

**Table 1:** **Details of gene expression datasets examined**

**Dataset**	**No. of classes**	**No. of genes**	**No. of samples**	**Negative samples**	**Positive samples**
Lung cancer	2	7129	96	86 primary lung adenocarcinoma samples	10 non-neoplastic lung samples
DLBCL	2	4026	47	24 GCB subgroup cases	23 ABC subgroup cases
Colon cancer	2	2000	62	40 tumor biopsy samples	22 normal biopsy samples
Prostate cancer	2	12,600	102	52 prostate tumor samples	50 non-tumor prostate samples
Leukemia	2	7129	72	25 AML bone marrow samples	47 ALL bone marrow samples

*Note*: DLBCL, diffuse large B-cell lymphoma; GCB, germinal center B-like; ABC, activated B-cell like; AML, acute myelocytic leukemia. ALL, acute lymphoblastic leukemia.

### Performance of genes selected by filter methods

First, the raw gene expression data in the five microarray datasets were normalized to zero mean and unit variance to account for the expression differences among genes. We then applied the filter methods, namely IG, gain ratio, reliefF, and correlation, for gene selection. The required number of genes selected cannot be determined using a common standard, but several hundred of genes are demonstrated to be sufficient to achieve high accuracy [18]. Therefore, different numbers of genes are selected for different filters with the number of genes ranging from 1 to 200. The LIBSVM classifier was used to evaluate the performance of the different numbers of selected genes.

**Figure 2:**
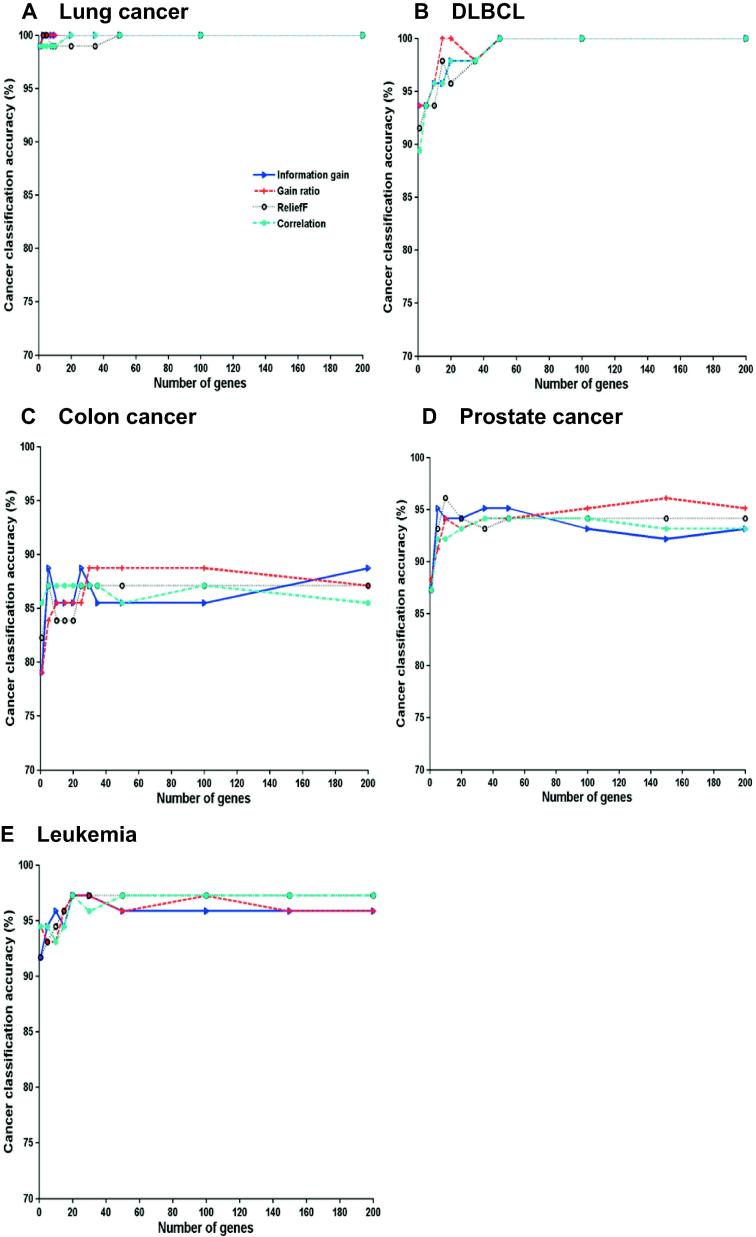
**Cancer classification performance using different filters**Classification accuracies plotted according to the number of genes selected using different filters, including information gain, gain ratio, reliefF, and correlation, are shown for lung cancer (**A**), DLBCL (**B**), colon cancer (**C**), prostate cancer (**D**), and leukemia (**E**), respectively. DLBCL, diffuse large B-cell lymphoma.

As shown in Figure 2, patterns for accuracies achieved with the numbers of selected genes appear to be different among the five different datasets tested. The highest accuracy for the five datasets differed. However, the overall trend was similar, indicating that the highest classification accuracy was commonly achieved using less than 50 genes. For instance, 3 genes were sufficient to reach a classification accuracy of 100% for lung cancer dataset, whereas the 100% of classification accuracy was achieved for the DLBCL dataset using 30 genes. The accuracies were maintained at a high level even when the gene number increased. For colon cancer, high performance was realized with no more than 10 genes, although a transient reduction in accuracy was found when the gene number was approximately 20.

Significant increases in the highest accuracy are not found with more than 50 genes selected, suggesting that considerable noise was present in the cancer datasets. Furthermore, slight and frequent fluctuations in accuracies were observed, owing to the presence of remaining redundant genes, and thus, further gene selection was required to obtain fewer significant genes.

### Performance of informative genes selected by hybrid methods

We next employed the wrapper method SVM to obtain informative genes. In addition, a 10-fold cross-validation of the LIBSVM classifier was adopted to evaluate the classification ability of the selected informative genes.

Considering that a small number of genes are sufficient to achieve high accuracy, 150 genes selected by the four types of filters were used as the input data for SVM. Since the top-ranked genes were more closely related to cancer classification, finally, the top 3 genes were selected for further cancer classification, using as few genes as possible.

Table 2 shows the classification accuracy of the four hybrid methods, *i.e.*, IG-SVM, gain ratio (GR)-SVM, reliefF-SVM, and correlation (Cor)-SVM. High accuracies were achieved using 3 genes only. The proposed method, IG-SVM, consistently performed better than the other hybrid methods. For instance, a classification accuracy of 100% was achieved for lung cancer and DLBCL using IG-SVM. Furthermore, both IG-SVM and Cor-SVM achieved 100% accuracy for the DLBCL dataset. Unlike that for lung cancer and DLBCL, the classification accuracy for colon cancer was relatively low, which was 90.32% for IG-SVM. Nonetheless the classification accuracy achieved in this study for colon cancer was still higher than that reported in another study (83.87%), which used the same number of selected genes by singular value decomposition and IG [18]. Since default settings were used for various tools, the possibility to achieve high accuracy with altered settings and selections can’t be ruled out.

**Table 2:** **Cancer classification accuracies (%) obtained based on the top 3 genes selected using hybrid methods**

**Dataset**	**IG-SVM**	**GR-SVM**	**ReliefF-SVM**	**Cor-SVM**
Lung cancer	**100**	98.96	98.96	98.96
DLBCL	**100**	97.87	95.74	**100**
Colon cancer	**90.32**	85.48	87.10	87.10
Prostate cancer	**96.08**	93.14	91.18	93.14
Leukemia	**98.61**	94.44	97.22	97.22

*Note*: Numbers in bold represent the highest accuracies achieved for the hybrid gene selection methods tested. DLBCL, diffuse large B-cell lymphoma; IG, information gain; GR, gain ratio; Cor, correlation; SVM, support vector machine.

Mao et al. [27] analyzed the same prostate cancer dataset and reported a mean classification accuracy of 95.10% by adopting randomization test (RT) as a gene selection method, which is lower than the accuracy achieved using IG-SVM in this study (96.08%). Using the aforementioned method, they obtained classification accuracies of 97.06% and 91.18% for the training and test sets for prostate cancer, respectively. As for the same leukemia dataset, the accuracies of 97.37% and 94.12% were reported for the training and test sets, respectively, using RT [27], which were below the accuracy we obtained for the entire dataset (98.61%).

### Disease association of selected informative genes

The important attributes derived from the microarray datasets are largely determined by the gene selection methods. In this study, the three datasets obtained from cancerous tissues with normal tissues as controls were further analyzed, including lung cancer, colon cancer, and prostate cancer datasets. The informative genes selected by the proposed IG-SVM method and their detailed description are listed in Table 3.

**Table 3:** **Informative genes selected using IG-SVM**

**Dataset**	**Selected genes**	**Probe set**	**IG value**	**Annotated gene**	**Annotation**
Lung cancer	F2968	M61906_at	0.377	*PIK3R1*	Phosphoinositide-3-kinase regulatory subunit 1
	F4530	U45973_at	0.322	*INPP5K*	Inositol polyphosphate-5-phosphatase K
	F5983	X61118_rna1_at	0.292	*LMO2*	LIM domain only 2

Colon cancer	F765	M76378_at	0.356	*CSRP1*	Cysteine and glycine rich protein 1
	F1423	–	0.315	*MYL9*	Myosin light chain 9
	F377	–	0.229	*GUCA2B*	Guanylate cyclase activator 2B

Prostate cancer	F6185	37639_at	0.675	*HPN*	Hepsin
	F7067	40436_g_at	0.366	*SLC25A6*	Solute carrier family 25 member 6
	F10234	41504_s_at	0.238	*MAF*	MAF bZIP transcription factor

*Note*: IG value of each gene in a dataset was calculated as described in the Methods section. All genes are ranked according to the IG values and the three selected informative genes are obtained using SVM.

In the lung cancer dataset, the top 3 genes selected are *PIK3R1*, *INPP5K*, and *LMO2*. *LMO2*, which encodes a LIM domain transcription regulator, is a proto-oncogene, and increased *LMO* expression has been reported in human lung tumors [28]. Similarly, *PIK3R1*, which encodes regulatory subunit 1 of phosphoinositide 3-kinase (PI3K) complex, has also been suggested as a lung cancer oncogene [29]. In a recent study, Deng and colleagues analyzed genomic variation in lung adenocarcinoma patients and found several PI3K family components including *PIK3R1* among the highly recurrent mutated genes, suggesting a critical role of PI3K signaling in the lung adenocarcinoma [30]. Notably, *INPP5K*, which encodes inositol polyphosphate-5-phosphatase K (also known as skeletal muscle and kidney enriched inositol phosphatase), was also selected in our study. *INPP5K* can hydrolyze PI(3,4,5)P3 generated by PI3 kinase to negatively regulate PI3K signaling [31]. Recent studies also identified *INPP5K* mutations in individuals exhibiting congenital muscular dystrophy [32], [33] or congenital cataract [34]. Although involvement of *INPP5K* in cancer progression has not been reported, *INPP5K* is located in a commonly deleted chromosomal region at 17p13.3 in various tumors [35]. In addition, a strong and significant reduction in *INPP5K* expression had been reported in a rat primary cell culture for endometrial carcinoma compared to the non-malignant endometrium [35]. These findings suggest that *INPP5K* could be a new tumor suppressor gene, which warrants further investigation.

*CSRP1*, *MYL9*, and *GUCA2B* were selected from the colon cancer dataset. *CSRP1* encodes a member of the cysteine-rich protein (CSRP) family, which may serve as an important biomarker of malignancy. *CSRP1* is inactivated in hepatocellular carcinoma [36], whereas *MYL9*, which encodes myosin light chain 9, shows prognostic significance in esophageal squamous cell carcinoma [37]. The third gene *GUCA2B* encodes guanylate cyclase activator 2B (also known as uroguanylin). Binding of uroguanylin to the receptor guanylate cyclase 2C may regulate salt and water homeostasis in the intestine and kidney [38]. It was observed that *GUCA2B* was significantly down-regulated in inflamed colonic mucosa of patients with inflammatory bowel disease (IBD) [39]. However, there is no direct evidence showing that these genes are associated with colon cancer. Therefore, their roles in colon cancer should be further investigated.

The three genes selected from the prostate dataset include *HPN*, *SLC25A6*, and *MAF*. *HPN*, which encodes the cell surface serine protease hepsin, is one of the most consistently overexpressed genes for prostate cancer, and hepsin protein expression is associated with the growth and progression of cancers, particularly prostate cancer [40]. In addition, some polymorphisms in the *HPN* gene might also be associated with the risk of developing prostate cancer [41]. The protein encoded by *SLC25A6* is a member of the mitochondrial ADP/ATP carrier subfamily of solute carrier protein genes. SLC25 family proteins play a role in cancer due to their decisive effect in the programmed cell death [42]. However, whether *SLC25A6* is related to prostate cancer still needs to be explored. The third gene *MAF* encodes a transcription factor. Defects in *MAF* can cause juvenile-onset pulverulent cataract as well as congenital cerulean cataract [43]. *MAF* is also a mediator of breast cancer bone metastasis [44]. Given the ubiquitous *MAF* expression in kidney, further studies are needed to investigate the relationship between *MAF* and prostate cancer. In short, there are several lines of evidence supporting that these genes may play important roles in cancer regulatory network, although their involvement in specific cancer types should be further examined.

## Conclusion

In this study, we proposed a hybrid method, IG-SVM to select informative genes for cancer classification. IG is a filter method that can efficiently eliminate numerous irrelevant features in high-dimensional gene expression data. The wrapper SVM method was used to further eliminate redundant genes based on 150 genes selected by filters. We finally obtained 3 informative genes, which served as the input for the LIBSVM classifier. By employing the tools for five gene expression datasets, we demonstrated better performance of the IG-SVM approach for cancer classification.

In summary, our study confirms that a few informative genes are sufficient to accomplish the accurate classification of tumor samples. Some of these selected informative genes have been shown to be associated with various cancers, whereas more evidence is needed for other genes selected, which may provide clues to functional studies and potential biomarker discovery. Given the small size of the datasets tested, the method proposed in this study need to be further validated in larger datasets.

## Authors' contributions

LG implemented the hybrid method and drafted the manuscript. MY participated in study design and coordination. XL and DH were involved in revising the manuscript. All authors read and approved the final manuscript.

## Competing interests

The authors declare that there are no potential conflicts of interest.
